# Development of a novel small antibody that retains specificity for tumor targeting

**DOI:** 10.1186/1756-9966-28-59

**Published:** 2009-04-30

**Authors:** Zi-Peng Zhen, Jie Zhang, Si-Yuan Zhang

**Affiliations:** 1 Key Laboratory of Transplant Engineering and Immunology, Ministry of Health, West China Hospital, Sichuan University, Chengdu 610041, PR China; 2 Linyi Normal University, Linyi, 276005, PR China; 3 West China Cancer Center/Laboratory of Signal Transduction and Molecular Targeting Therapy, West China Hospital, Sichuan University, Chengdu 610041, PR China

## Abstract

**Background:**

For the targeted therapy of solid tumor mediated by monoclonal antibody (mAb), there have different models of rebuilding small antibodies originated from native ones. Almost all natural antibody molecules have the similar structure and conformation, but those rebuilt small antibodies cannot completely keep the original traits of parental antibodies, especially the reduced specificity, which gravely influences the efficacy of small antibodies.

**Methods:**

In this study, authors developed a novel mimetic in the form of V_H_FR1_C-10_-V_H_CDR1-V_H_FR2-V_L_CDR3-V_L_FR4_N-10_for a parental mAb induced with human breast cancer, and the mimetic moiety was conjugated to the C-terminal of toxicin colicin Ia. The novel fusion peptide, named protomimecin (PMN), was administered to MCF-7 breast cancer cells to demonstrate its killing competency *in vitro *and *in vivo*.

**Results:**

Compared with original antibody-colicin Ia (Fab-Ia) and single-chain antibody-colicin Ia (Sc-Ia) fusion proteins, PMN retained the targeting specificity of parental antibody and could specifically kill MCF-7 cells *in vitro*. By injecting intraperitoneally into BALB/c athymic mice bearing MCF-7 tumors, with reduced affinity, PMN significantly suppressed the growth of tumors compared with control mice treated by toxicin protein, Fab-Ia protein, Sc-Ia protein or by PBS (*p *< 0.05).

**Conclusion:**

This novel mimetic antibody retained original specificity of parental antibody, and could effectively guide killer moiety to suppress the growth of breast cancer by targeted cell death.

## Background

Targeted therapy with maximal effectiveness and minimal adverse effects is the ultimate goal for treatment of solid tumors [1,2]. Since the development of hybridoma and monoclonal antibody (mAb) technology [3,4], antibody therapy has emerged as the choice for targeted therapy for solid tumors because of the specific affinity of the antibody for the corresponding antigen, owing to the presence of six complementarity-determining regions (CDRs) in the variable domains of the heavy chain (V_H_) and that of light chain (V_L_) [3,5]. However, although native antibodies have the highest specificity and affinity for antigens, they also have large molecular structures and the potency of penetrating into the core area of solid tumors cannot reach to the extent that scientists expect because of the "binding barrier"[6]. Single-chain Fvs (scFvs) contain the specificity of the parental antibody molecules, but they readily form aggregations [7]. Overlooking the synergistic antigen recognition relationship between V_H _and V_L_, artificially rebuilt single-domain antibodies or micro-antibodies cannot completely keep the specificity and affinity of parental antibody [8,9].

**Figure 1:**
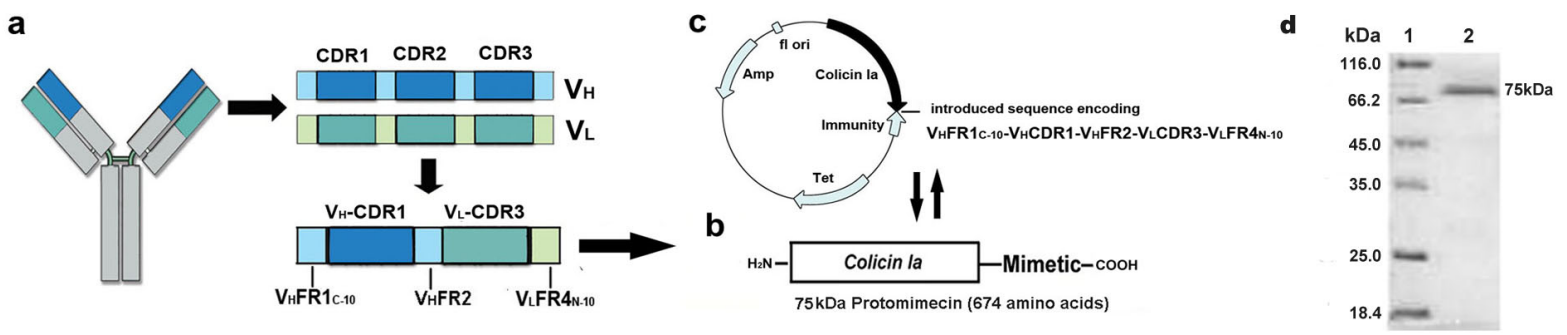
**Schematic diagram for the process of constructing the small antibody and the fusion peptide**. (a) The small antibody (the mimetic moiety) was composed of **V_H_FR1_C-10_-V_H_CDR1-V_H_FR2-V_L_CDR3-V_L_FR4_N-10_**. (b, c) The mimetic was conjugated to the C-terminal of wild-type colicin Ia to construct the conjugated peptide, named protomimecin (PMN). (d) The 15% SDS- PAGE migration map of the fusion peptide PMN.

We proposed that the essential interface of antibody-antigen binding constrained by the molecular forces between V_H _and V_L _[10,11]. For original antibody molecules, the constraint force derives from the 3-Dimension conformation of antibody molecules. Our small antibody was constructed in the following form: V_H_FR1_C-10_-V_H_CDR1-V_H_FR2-V_L_CDR3-V_L_FR4_N-10 _(Fig. 1a). Antigen recognition by intact antigen-binding fragment (Fab) of immunoglobulin (Ig) is synergistically produced by all six CDRs in both V_H _and V_L _domain, CDR3 is located in the center of the antigen-recognition interface of the parental antibody and should be contained within the internal portion of the small antibody [12]. Another CDR domain selected was V_H_CDR1 normally the closest to CDR3, which formed the synergistic interface with CDR3 for antigen-recognition [8,9]. The V_H_FR2 segment linked the two CDRs and contains the least hydrophobic amino acid (aa) residues, increasing the water solubility of the mimetic complex. Finally, V_L_FR4_N-10 _and V_H_FR2 supported CDR3 to form the projected loop conformation, and the V_H_CDR1 loop was restrained on both sides by V_H_FR2 and V_H_FR1_C-10 _forming the other loop conformation. These selected components of the mimetic are original and not changed or substituted from the parental antibody. Guided by these reasons, we proposed that the construct of mimetic kept specificity similar to that of parental antibody (Fig. 1a).

In the present study, we constructed the small antibody consisting of V_H_FR1_C10_-V_H_CDR1-V_H_FR2-V_L_CDR3-V_L_FR4_N10 _conjugated in-line, as a mimetic molecule for a natural monoclonal IgG against human breast cancer cell envelope antigen c-erbB-2 [13,14]. The mimetic was then conjugated to the C-terminal of colicin Ia, a 70-kD member of the E1 colicin family of channel-forming bacteriocins that are bactericidal to *Escherichia coli *(*E. coli*) to obtain a fusion protein, named protomimecin (PMN; Fig. 1b, c), which enable us to demonstrate the ability of the mimetic to target cancer cells bearing specific surface antigens. Colicin Ia kills target cells by forming a voltage-activated channel in the cell membrane of target cells mediated by its C-terminal 175-residues, channel-forming domain which contains the killing competency of "one molecule, one kill" [15,16]. We demonstrated that PMN could effectively kill MCF-7 cells *in vitro *and suppress the growth of MCF-7 tumors *in vivo*. Based on our preliminary results, this novel model of reconstructing small antibodies may be further developed for targeted therapy of tumors.

## Methods

### Cell lines and cell culture

The hybridoma cell line HB-8696 was purchased from ATCC and grown in Dulbecco's modified Eagle Medium (DMEM) and fortified with penicillin-streptomycin (100 U/ml, 100 μg/ml respectively) and 10% fetal bovine serum (FBS). Medium was changed every 2–3 days. The breast cancer cell lines, Zr-75-30 and MCF-7, and the Burkitt's Lymphoma cell line, Raji (obtained from the Laboratory of Transplant Immunology and the Department of Laboratory Medicine, Division of Clinical Immunology, West China Hospital) were grown in RPMI 1640 medium containing double antibiotics and 10% FBS. Medium was changed every 2–3 days. All cell lines were incubated at 37°C in 5% CO_2 _incubator (Sanyo Electro. Biomed. Japan).

### The preparation of parental antibody 520C5 and toxicin colicin Ia

HB-8696 murine hybridoma cells were grown to a density of 10^7 ^cells/ml. Under sterility and 4°C, the cells were removed from the medium by centrifugation at 1000 rpm, and the supernatant (containing the original mAbs 520C9 that are the parental antibody of the mimetic peptide molecules) was further purified by centrifugation at 10,000 g. The following purification procedure was done according to purification kit' protocol (Millipore). The purified antibodies were stored at -20°C for subsequent experiments.

The p^SELECT^-1 plasmids (from the Key Laboratory of Transplant Engineering and Immunology, Ministry of Health, West China Hospital, Sichuan University, China) that contain the colicin Ia gene and the reversed direction immunity protein gene of colicin Ia were transformed into competent TG1 cells. Then spread those TG1 cells on FB agar medium containing 25 μg/ml ampicillin and cultivated at 37°C for 12–16 hours under humidity and screening TG1 cells containing p^SELECT^-1 plasmids, and the positive clones were selected to cultivate rotatorily at 180 rpm in 2 ml FB medium containing 50 μg/ml ampicillin under the same condition as above mentioned for overnight, then carefully dumped to 60 ml FB medium for continuous cultivation for 5–6 hours. Until the total volume of medium reached 8 × 600 ml and the OD for TG1 cells reached 0.5 under same culture condition, centrifuged those cells at 6,000 g for 17 minutes under 4°C, and resuspended precipitate in 60–80 ml borate buffer (50 mM borate buffer, pH 9.0, with 2 mM EDTA) containing 0.5 mM phenylmethylsulfonyl fluoride. The cells were sonicated and debris removed by centrifugation for 90 min at 75,000 *g *under 4°C. Nucleic acids were precipitated by addition of 1/5 volume streptomycin sulfate (25%). Supernatants were dialyzed against borate buffer for 12 hours (changing the buffer every 5–6 hours) at 4°C then applied to the ÄKTA™ prime protein purification system (2.5 × 12 cm CM-Sepharose column, Amersham Pharmacia Biocech). Proteins were recovered at 4°C by gradient elution with 0.1, 0.2 and 0.3 M NaCl in borate buffer and collected in 0.5 ml fractions. The harvested colicin Ia was dialyzed against PBS (pH 7.4–7.5) for 12 hours at 4°C, and stored at -80°C freezer for subsequent experiments.

### The scanning of V_H _and V_L _domain DNA sequences of original antibody

V_H _and V_L _domain genes for mAb A520C9 IgG were isolated from HB-8696 mouse hybridoma cell. Total RNA was extracted and amplified by RT-PCR (Takara RNA PCR Kit (AMV Ver.3.0)) using the following primers: **H-chain**: 5'-ACTAGTCGACATGGCTGTCYTRGBGCTGYTCY TCTG-3'and 5'-CCCAAGCTTCCAGGGRCCARKGGATARACWGRTGG-3'; **L-chain**: 5'-GGGAATTCATGGAGACAGACACACTCCTGCTAT-3'and 5'-CCCAAGCTTACTGGA TGGTGGGAAGATGGA-3', purified RT-PCR products were ligated into the plasmids pMD18-T, purchased from Takara. The DNA sequences of plasmids were isolated and analyzed to determine the genes of V_H _and V_L _domains of mAb.

### Amino acid sequences of peptides from parental antibody

The aa sequences of all six CDRs in the parental antibody 520C9 Fab are:

V_H_: H_2_N-EMQLVESGPEVKKPGASVKVSCKASGYTFT**NYGMN**WVRQAPGQGLEWM G**WINTYTGQSTYADDFKE**RVTMTTDTSTSTAYDMLRSLRSDDTAVYYCAR**RFGFAY**WQ GTLVVSS-COOH (bold letters represent CDR domain)

V_L_: H_2_N-DIQMTQSPSSLSASVGDRVTITC**RASQDIGNSLT**WYQQKPGKTPKLLIY**ATS SLDS**GVPSRFSGSGSGTDFTFTISSLQPEDIATYYC**LQYAIFPYT**FGQGTRLEIK-COOH (bold letters represent CDR domain)

The sequence of the single-chain Fv (ScFv) for the parental antibody 520C9 is:

V_H_-(GGGGS)_3_-V_L _[17]

The sequence of the V_H_FR1_C-10_-V_H_CDR1-V_H_FR2-V_L_CDR3-V_L_FR4_N-10_mimetic of the parental antibody is:

H_2_N-SCKASGYTFTNYGMNWVRQAPGQGLEWMGLQYAIFPYTFGQGTRLEIK-COOH

### Preparation of the mimetic moiety and conjugated peptide

The DNA sequences for the V_H_FR1_C-10_, V_H_CDR1, V_H_FR2, V_L_CDR3 and V_L_FR4_N-10 _regions of the 520C9 Fab were conjugated to follow position I626 of colicin Ia by double-stranded oligonucleotide mutagenesis (QuickChange kit, Stratagene) using the p^SELECT^-1 plasmid that contains the colicin Ia gene and the reversed direction immunity protein gene of colicin Ia to form colicin Ia-V_H_FR1_C-10_-V_H_CDR1-V_H_FR2-V_L_CDR3-V_L_FR4_N-10 _(Fig. 1). The oligonucleotides used contained the desired mutations for **SCKASGYTFTNYGMNWVRQAPGQGLEWMGLQYAI FPYTFGQGTRLEIK **were 5'-GCG AAT AAG TTC TGG GGT ATT **TCC TGC AAG GCT TCT GGT TAC ACC TTT ACC **TAA ATA AAA TAT AAG ACA GGC-3', 5'-GCT TCT GGT TAC ACC TTT ACC **AAC TAT GGA ATG AAC TGG GTG CGA CAG GCC **TAA ATA AAA TAT AAG ACA GGC-3', 5'-ATG AAC TGG GTG CGA CAG GCC **CCT GGA CAA GGG CTT GAG TGG ATG GGA CTA **TAA ATA AAA TAT AAG ACA GGC-3', 5'-GGG CTT GAG TGG ATG GGA CTA **CAA TAT GCT ATT TTT CCG TAC ACG TTC GGC **TAA ATA AAA TAT AAG ACA GGC-3' and 5'-ATT TTT CCG TAC ACG TTC GGC **CAA GGG ACA CGA CTG GAG ATT AAA **TAA ATA AAA TAT AAG ACA GGC-3' (boldface triplets represent inserted sites).

Plasmids containing inserted DNA sequences were transformed into competent TG1 *E. coli*, and cells were grown in FB medium containing 50 μg/ml ampicillin. The procedures of cultivating TG1 cells and purifying conjugated peptides were the same as that of preparing colicin Ia protein.

### *In vitro *killing activity, Immunolabeling and affinity assays

ZR-75-30, MCF-7, and Raji cells were grown in the Falcon 3046 six-well cell culture plates (Becton Dickinson Co.) under the same condition as that of above described. 24 hours later, 5–125 μg/ml PMN, wild type colicin Ia (wt Ia), parental antibody-colicin Ia fusion protein (Fab-Ia), single-chain antibody-colicin Ia fusion protein (Sc-Ia) (CL(Xi'an) Bio-scientific) and nonrelative control protein, low molecular weight marker protein (LWMP, purchased from Takara) were respectively added to the cell culture wells. After co-incubating for 24 hours, the living and dead cells were stained with 50 nM acridine orange and 600 nM propidium iodide and staining was imaged using a digital data collection system under an inverted fluorescent microscope (IX-71, Olympus) using U-MWU2, U-MNB2 and U-MNG2 filters. For the comparison of killing competency presented by those agents with each other, we selected five image fields to respectively count the number of dead and living cells in every culture well after 24, 48 and 72 hours.

MCF-7 cell were grown in 1640 medium for 72 h, fixed in 10% paraformaldehyde for 40 min at room temperature, then 100 μl fixed cells (10^6^/ml) were incubated with 10 μl PBS, LWMP, Fab, Sc (CL(Xi'an) Bio-Scientific) and PMN respectively with different concentration (10^2^-10^-1^nM) for 1 hr at 37°C, then incubated with parental antibody for 40 min at 37°C and fluorescein isothiocyanate (FITC) -labeled second antibody (Pierce) for 30 min at 37°C. After incubating with DAPI for 25 minutes at 37°C, the mean fluorescent intensity of per 1,500 cells was measured by BD FACSCanto Flow Cytometer (BD Biosciences). For concentration-dependent inhibitory experiments against the killing activity of PMN, different concentrations of either parental A520C9 mAbs, or synthetic V_H_FR1_C-10_-V_H_CDR1-V_H_FR2-V_L_CDR3-V_L_FR4_N-10 _(South West University) were added with PMN (75 μg/ml) to incubate with MCF-7, Zr-75-30 or Raji cells, respectively (10^2^-10^-1^nM), then living and dead cells were counted with 0.2% Trypan blue under an inverted microscope (IX-71, Olympus).

The MCF-7 cells were grown and fixed as the above-mentioned procedure. Then original antibodies (OAbs) and the mimetic peptides were diluted to 100, 10, 1 and 0.1 μmol/L by PBS (pH7.45), respectively. The indirect enzyme-linked immunosorbent assays (ELISA) were introduced to analysis the relative affinity of the mimetics and OAbs to antigens. The value of absorbance at 490 nm wavelength was inspected by microplate reader (Bio-Rad), which was used to determine the concentration of the OAbs and the mimetics when the saturation of Abs to antigens reached to one percent. The relative affinity was compared between OAbs and the mimetics at 50% saturation of Abs to antigens.

### *In vivo *activity and the biodistribution of PMN

MCF-7 cells were grown under the same condition as that of above described, and collected by centrifugation at 1,000 rpm. Cells were resuspended in FBS-free medium at a concentration of 10^8 ^cells/ml. Twenty-five 4–5-week-old female BALB/c athymic nude mice weighing 16–20 g were purchased from the Experimental Animal Center of West China Hospital. Before implanting tumor cells, mice were allowed to acclimatize for 3 days. A total of 6–7 × 10^7 ^MCF-7 cells were subcutaneously (s.c.) implanted into the left armpit of mice. Tumor growth was monitored daily until the average sizes of tumors reached 5 × 5 × 5 mm, then randomly separated those mice to the treatment group (PMN group; n = 5), wild type colicin Ia group (wt Ia group; n = 5), Fab-Ia group (n = 5), Sc-Ia group (n = 5) and the PBS control group (PBS group; n = 5), and the treatment course began. The PMN group was treated with intraperitoneal (i.p.) injection of PMN at 1,200 μg/mouse/day (400 μg/8 hours, tid; n = 5). The wt Ia group, Fab-Ia group, Sc-Ia group and the PBS group were injected with wt Ia protein, Fab-Ia protein, Sc-Ia protein (400 μg/8 hours, i.p. tid; n = 5) and PBS (450 μl/8 hours, i.p. tid; n = 5), respectively. Animals had free access to standard food and water throughout the treatment course. After 14 days, all mice were sacrificed to collect tumors and organs for weighing and for histopathological inspection.

150 μg PMN proteins labeled by FITC (EZ-labeled FITC protein labeling kit, pierce) were ip injected into BALB/c mice (n = 5), weighing 16–20 g, inoculated MCF-7 cells at armpit for 2 weeks. 2.5 hours later, the mice were fastened supinely on a black board under ether inhalation. When the *in vivo *inspections completed, the mice were sacrificed and the tumors and vital organs were sectioned. The images were observed with the LT-99D2 Illumatool Dual Light System (excitation 470 nm, emission 515 nm, Lightool Research) and recorded by a built-in camera.

### Assessment of toxicity of PMN

Kunming normal mice (purchased from Experimental Animal Center of West China Hospital, Sichuan University, China), weighing 15–25 g were injected with either PMN (100–2,500 μg/mouse/day, n = 5) or PBS (n = 5) intraperitoneally each day. After 3 weeks of administration, mice were sacrificed for histopathological inspection and blood samples were collected for indirect enzyme-linked immunosorbent assay (ELISA) to screen potential antibodies.

The Institutional Animal Care and Use Committee of Sichuan University and Project of Sichuan Animal Experiment Committee (license 045) approved the animal use and *in vivo *experiments.

### Electrophoresis

0.9% agarose electrophoresis was applied to authenticate the reconstructed plasmids and 15% sodium dodecyl sulfate polyacrylamide gel electropheresis (SDS-PAGE) was applied to authenticate the harvested protein, respectively.

### Statistical analysis

SPSS version 11.0.1 for Microsoft Windows was used for statistical analysis. Two-tailed *t*-tests were performed using GraphPad Prism for Windows version 4.00. *P *< 0.05 was considered to be a statistically significant difference.

## Results

### Production and purification of PMN

Plasmids containing the colicin Ia gene and the reversed direction immunity protein gene of wt Ia protein were used to conjugate signal-moiety with wt Ia (Fig. 1c). We conjugated the 48-aa residues to the C-terminal of wt Ia by five mutation steps, with the same PCR reaction conditions (95°C, 35 sec for denaturation; 53°C, 70 sec for annealing; 68°C, 17 min for elongation; which repeated 18 times). Plasmid migration in agarose electrophoresis (0.9%) was applied to confirm transmutated plasmid at each step (data not shown). After the last round of PCR, the harvested plasmid was transformed into competent TG1 *E. coli *to produce the PMN protein.

PMN protein was eluted with 0.2 M NaCl borate buffer. The original molecular weight of wt Ia is ~70 kDa and, with the addition of the 48-aa residues (approximately 5.3 kDa), the molecular weight of PMN is ~75 kDa, which was confirmed by SDS-PAGE migration image (Fig. 1d).

### *In vitro *killing activity and specificity of PMN

**Figure 2:**
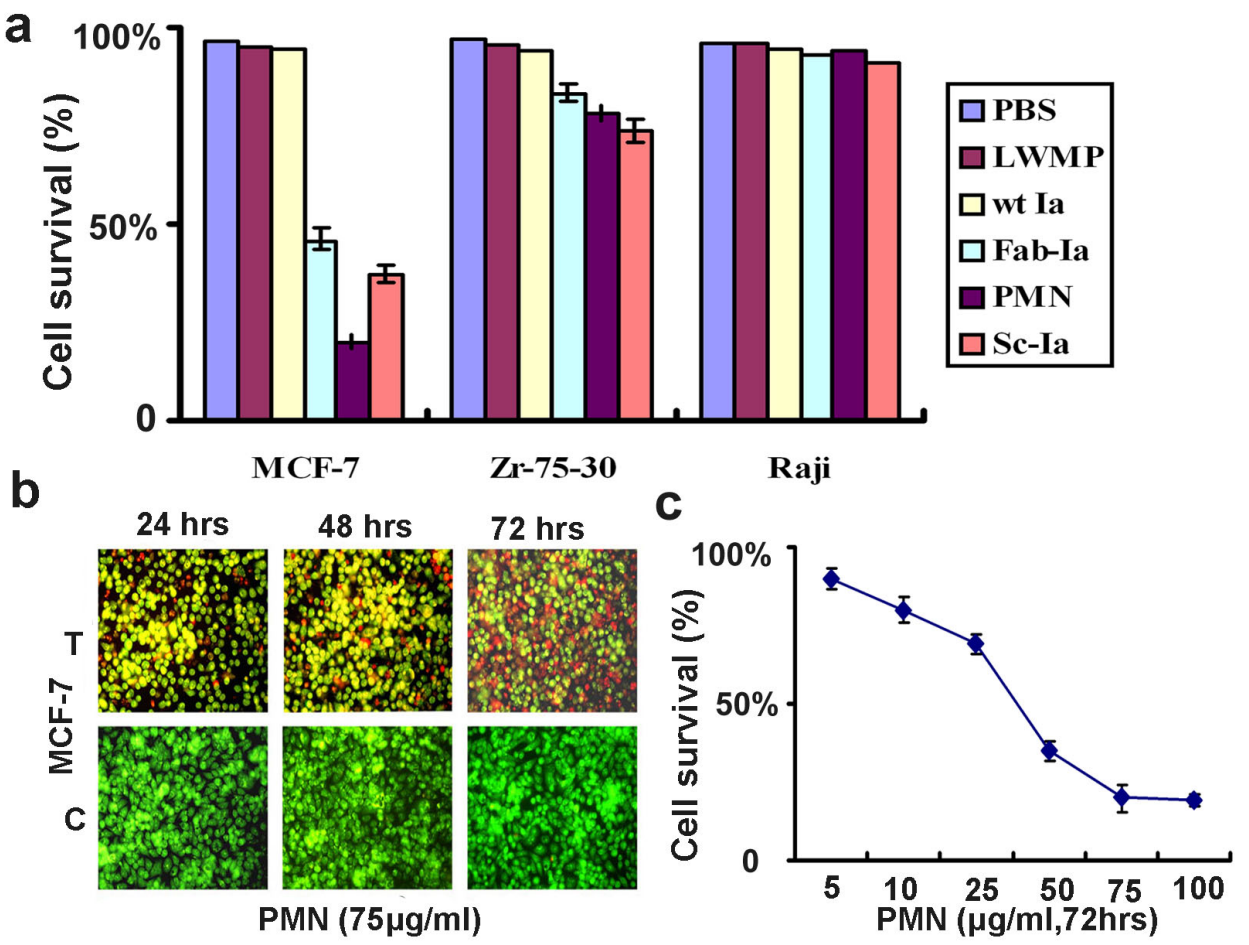
***In vitro *killing activity assays of PMN**. (a) Killing effects of PBS, non-relative LWMP, wt Ia, Fab-Ia, PMN and Sc-Ia on MCF-7, Zr-75-30 and Raji cells lines. LWMP, low molecular weight marker protein; wt Ia, wild-type colicin Ia; Fab-Ia, Fab segment from original antibody-colicin Ia fusion peptide; PMN, protomimecin; Sc-Ia, ScFv segment from original antibody-colicin Ia fusion peptide. (b) MCF-7 breast cancer cells were incubated with 75 μg/ml PMN for 24, 48 and 72 hrs, respectively. And tumor cells were stained with acridine orange (green color) and propidium iodide (red color). Red spots, dead cell mass; Green spots, live cell. After co-incubation for 72 hrs, approximately 80% of all MCF-7 cells had died (upper panel). T, PMN-treated group; C, control group treated with PBS. (c) Cytotoxicity of different concentration of PMN against MCF-7.

Against MCF-7 cells, PMN molecules presented dramatic killing competency. Compared with Fab-Ia and Sc-Ia, who both presented obvious killing competency to MCF-7 cells, the killing competency of PMN molecule to MCF-7 cells was significantly superior to them (p < 0.05, Fig. 2a). The killing activity of PMN presented time- and concentration-dependent characteristics. Of these cells, about 70–85% of the MCF-7 cells were killed within 48–72 hours after exposure to the PMN at concentration 75 μg/ml (p < 0.001; Fig. 2b) and 72 hours after incubating, PMN presented no obvious killing effect on MCF-7 cells at concentration 5 μg/ml, but killed 80% of cells at concentration 75 μg/ml (Fig. 2c). Neither wt Ia protein nor the nonrelative LWMP could kill MCF-7, Zr-75-30 and Raji cells up to the maximal tested concentration at any time points (125 μg/ml). 72 hours co-incubation of Zr-75-30 and Raji with Fab-Ia, Sc-Ia, PMN and LWMP peptide molecules at any concentration did not significantly affect the viability of these cells relative to untreated control (Fig. 2a).

**Figure 3:**
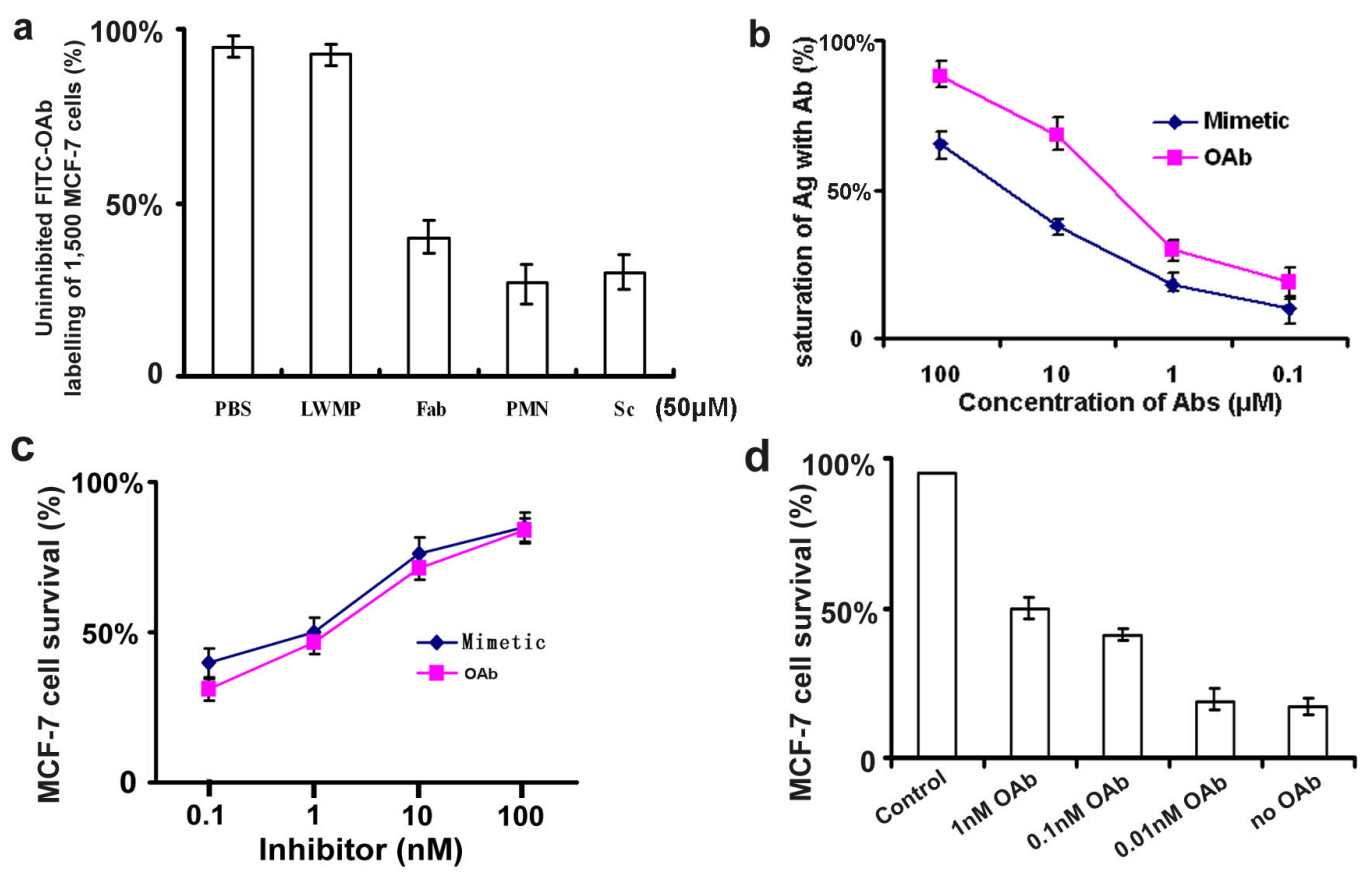
**The competition ability of synthetic V_H_FR1_C-10_-V_H_CDR1-V_H_FR2-V_L_CDR3-V_L_FR4_N-10_**. (a) Fixed MCF-7 cells were incubated with PBS, LWMP, Fab, PMN and Sc-Fv peptides (50 μM) and DAPI (50 ng/ml) prior to flow cytometry. LWMP, low molecular weight marker protein; Fab, Fab segment from original antibody; PMN, protomimecin; Sc-Fv, ScFv segment from original antibody. (b) The relative affinity of V_H_FR1_C-10_-V_H_CDR1-V_H_FR2-V_L_CDR3-V_L_FR4_N-10 _peptides and OAbs to antigen. OAb, original antibody. (c) Concentration-dependent inhibition of different concentration of synthetic mimetic antibody or OAb against 75 μg/ml PMN. (d) MCF-7 cell survival ratio of the inhibition activity of OAb against PMN (75 μg/ml). OAb, original mAb antibody A520C9.

We assessed the antigen-recognition capabilities of PMN, Fab, Sc-Fv, LWMP and wt Ia peptides against MCF-7 cell by competition with the parent antibody. The results indicated that the V_H_FR1_C-10_-V_H_CDR1-V_H_FR2-V_L_CDR3-V_L_FR4_N-10 _mimetic had nearly the same extent effect on blocking binding of the parent antibody as Fab and Sc-Fv peptides (Fig. 3a). The concentration of the mimetic peptides that could induce 50% saturation of antigen was about 10~15% that of OAbs (Fig. 3b). The killing activity of PMN molecules against MCF-7 cells could be inhibited up to 90% by increasing concentrations of OAbs or synthetic V_H_FR1_C-10_-V_H_CDR1-V_H_FR2-V_L_CDR3-V_L_FR4_N-10 _mimetic molecules (Fig. 3c, d).

### *In vivo *activity and the biodistribution of PMN

**Figure 4:**
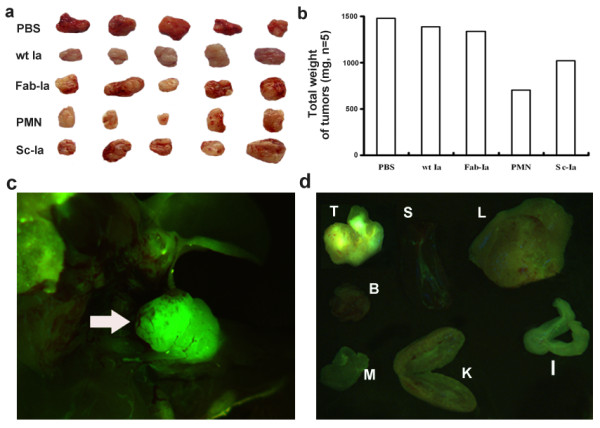
***In vivo *killing competency and the biodistribution of PMN**. *In vivo *killing competency was compared with PBS, wt Ia, Fab-Ia and Sc-Ia in BALB/c athymic immunocomposed mice bearing MCF-7 tumors. (a) The tumors of mice were collected after 2-week administration. (b) The weights of each individual tumor were added together and the total weights were compared between groups. Compared with PBS, wt Ia, Fab-Ia and Sc-Ia, PMN could significantly suppress the growth of MCF-7 tumors (p < 0.05). PMN, protomimecin; wt Ia, wild-type colicin Ia; Fab-Ia, Fab segment from original antibody-colicin Ia fusion peptide; Sc-Ia, ScFv segment from original antibody-colicin Ia fusion peptide. (c) Fluorescence images of tumor (white arrow) in BALB/c mice traced by FITC-labeled PMN. The green fluorescence represented the location of FITC-labeled PMN protein. (d) Fluorescence images of incised tumor and vital organs from BALB/c mice traced by ip injecting FITC-labeled PMN. The green fluorescence showed the biodistribution of FITC-labeled PMN. T, tumor; S, spleen; L, liver; B, brain; M, muscle; K, kidney; I, intestine.

PMN, Fab-Ia and Sc-Ia agents were administered to tumor-bearing BALB/c nude mice at 1,200 μg/mouse/day (400 μg/8 hours, i.p. tid). Compared with the control groups treated by PBS, wt Ia, Fab-Ia and Sc-Ia agents, PMN effectively suppressed the growth of MCF-7 tumors *in vivo *(Fig. 4a). At the end of the consecutive 14-day treatment, the total tumor weight was significantly low in the PMN treatment group by about 45% compared with the other control groups (*p *< 0.05; Fig. 4b).

The fluorescence images revealed the targeting accumulation in MCF-7 tumor location within 2.5 hours after intraperitoneal injection (Fig. 4c). There were no same extent accumulations found in other vital organs except the intestine (Fig. 4d).

### The bio-safe assessment of PMN

Those immunocompromised mice bearing tumors and those normal Kunming mice both treated by PMN remained health and gained body weight during the experimental course. Indirect ELISA found no detectable antibodies against respective epitopes in normal mice after 3 weeks treatment with different concentration PMN. The histopathological detection found no microscopic evidences of necrosis, inflammation or lymphocyte infiltration in the livers, spleens, kidneys and intestines from normal mice (data not shown).

### Histopathological analysis

**Figure 5:**
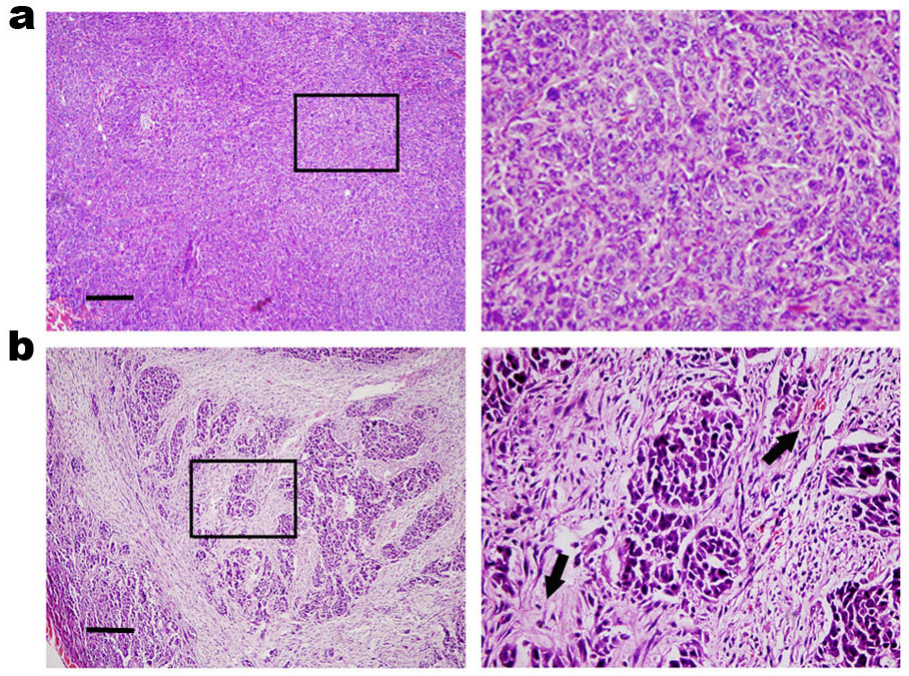
**Histopathological staining revealed numerous fibrous foci (black arrow) in the tumors from the treated group with PMN (b), which were not seen in the other control groups (a)**. PMN, protomimecin. Scale bar, 50 μm.

We found numerous fibrous foci in tumors from the PMN-treated group (Fig. 5b), which were not observed in the control groups' tumors (Fig. 5a). No microscopic evidence of metastasis, necrosis, inflammation or lymphocyte infiltration was detected in the livers, spleens, kidneys and intestines from BALB/c mice (data not shown).

## Discussion

In this study, we introduced a new model of reconstructing small antibody for targeted therapy of solid tumors, considering that the proper CDRs loops could not automatically form in the medium without the constraint force, which made the working conformation for the antigen-antibody interaction could not maintained [18]. We added two frameworks without any residue substitutions from original ones to both ends of two selected CDRs to restrain their conformation for the following three reasons. First, sustaining both CDRs as protruding loop structures should increase the probability of accessing target epitopes of specific antigens [19]. Second, for the mimetic, constraining the conformation of CDRs should reduce the probability of forming improper conformations and increase the efficiency of antigen-recognition by the proper conformation [8,20]. Third, the interactions among the framework moieties of the mimetic molecules should most effectively simulate the same kind of constraint force that exists among the frameworks of original antibody molecules [8,11,20]. Guided by those reasons, we posited that adding two restricted frameworks, with one at each end, could further constraint the conformation of V_H_CDR1 and V_L_CDR3 loops in the mimetic. Based on previous studies [10], we proposed that the length of the two framework fragments should be at least 10-aa. Therefore, the C-terminal 10-aa residues of V_H_FR1 (V_H_FR1_C-10_) were attached to the N-terminal of V_H_CDR1 and the N-terminal 10-aa residues of V_L_FR4 (V_L_FR4_N-10_) were attached to the C-terminal of V_L_CDR3 to form V_H_FR1_C-10_-V_H_CDR1-V_H_FR2-V_L_CDR3-V_L_FR4_N-10 _that could produce the constraint force by which the proper CDR1 and CDR3 loops formed.

Our findings suggested the proper loops of V_H_CDR1 and V_L_CDR3 were sustained in our small antibody model. The competition test to assess inhibition of PMN binding to specific antigens by parental antibody and synthetic mimetic demonstrated that the mimetic model without any substitution from original antibody contained the specificity (Fig. 3), and the *in vitro *results demonstrated this model kept the same extent specificity as Fab and ScFv did (Fig 2a, 3a). The antigen-recognizing moiety of the conjugated reagent could discern the specific antigens on the breast cancer cell membrane, and guide the colicin-derived moiety to form a transmembrane ion-channel and ultimately killed the target cells [15]. Owing to the absence of the specific antigen in the Raji cells, the mimetic moiety should be unable to interact with these cells, so they survived the lethal effects of PMN molecules (Fig. 2a). Not only *in vitro *condition, but also *in vivo *condition PMN could present its competency of recognizing specific antigen on target cells and killing them, which was also confirmed by our experiments finding no obvious effects on growth of MCF-7 tumor treated by wt Ia protein (Fig. 4).

Compared to its parental antibody, this small antibody reconstructed following the novel way kept only part affinity to antigen (Fig. 3b). And *in vivo *results certificated PMN molecules could penetrate into the core area of solid tumors. However, the increased affinity could not improve the penetration into solid tumors [21]. Previous studies had already confirmed that the highest affinity scFv mainly arrested in the perivascular region of solid tumors, whereas the lowest affinity scFv harbored the most uniform biodistribution throughout the tumors [6]. This novel small antibody contained only 10~15% original affinity, which assigned the mimetic increased penetration and kept the specificity (Fig. 4, 5). Considering the synthetic relationship between specificity and affinity in the procedure of interacting of antibody to antigen [1,2,7], under the condition of keeping original specificity, maybe the reduced affinity of those rebuilt small antibodies could give a more better solution to the "binding-barrier" of solid tumors than only keeping the single specificity or affinity.

*In vitro *results indicated that the Fab and Sc-Fv signals could guide the "killing moiety" to kill breast cancer cells, but those phenomena could not be re-presented *in vivo*. It was suggested that the solid tumors, especially malignant tumors have interstitial fluid pressure in their tissues because of the eugonic state, which prevents the diffusion of any forms of treatment medicines into the core area of solid tumors, especially those large peptide molecules such as native antibody Fab and ScFv segments [22,23]. By pathological staining, we found numerous fibrous foci in the core area of the tumors from treated mice, which were not inspected on tumors from the control animals including the Fab-Ia and Sc-Ia groups (Fig. 5), indicating that PMN molecules could efficiently penetrate into the core area of solid tumor and kill target cells. Previous studies on exnograft MCF-7 tumors show no evidence of metastasis and no obvious fibrosis, which is consistent with our results [24] (Fig. 5a). We observed that the parenchyma of treated tumors presented numerous areas of embedded fibrous tissue, indicating that the parenchyma was substituted by fibers and other connective tissue components after necrosis (Fig. 5b). Compared with the control group tumors, which showed much parenchyma with little abnormal connective tissue, the pathological difference between the tumors from PMN-treated and control groups may be more important than just the weight difference between the groups, although the total tumor weight difference from groups was significant (*p *< 0.05) after 2-week treatment.

Furthermore, we found expression intensity of c-erbB-2 antigen was higher on Zr-75-30 than on MCF-7 cells, but those reagents including PMN, Fab-Ia and Sc-Ia fusion peptides produced no obvious effects on Zr-75-30 cells *in vitro *(Fig. 2a), which was also found in previous studies, showing that the same antibody conjugated to toxins or other reagents could not always present the same killing competency in all tested cell lines [14,15,25,26]. However, for PMN, Fab-Ia and Sc-Ia, we believed that a "binding barrier" [6], originating from intensive interactions between the antigen and antibody, prevented the α-helix hairpin of the channel-forming domain of colicin Ia from approaching the plasma membrane [15,16], which blocked the killing effect. Furthermore, the mimetic generally kept only 10~15% affinity of parental antibody to antigen (Fig. 3b). More importantly, the c-erbB-2 membrane glycoprotein is a complicated antigen, and contains different epitopes on its surface. Although almost all of those breast cancer cells express the same antigen c-erbB-2, the precise epitope and the specific targeting site may be different to each other. However, the precise reason for the reduced efficacy to other breast cancer cell lines remains to be resolved.

The PMN peptide molecule mainly consists of conlicin Ia (Fig. 1). The E1 colicin family protein are produced by *E. coli *and permanently existed in live beings. And because of the parasitism of *E. coli *in intestine, which means this peptide is an immunological tolerant protein for those parasitifers. Our bio-safe assessment assays demonstrated the safety of this novel fusion peptide, showing all the experimental animals gained body weight during experiments, and no microscopic evidences of metastasis, necrosis, inflammation and lymphocyte infiltration were detected in liver, kidney, intestine, lung and spleen from groups treated by PMN. Those results suggested the *in vivo *bio-safety of the novel peptide could be assured. But the potential toxicity of the toxin-mimetic conjugated peptide remains to be investigated before using in human.

## Conclusion

The present research confirmed that the novel mimetic maintained the specificity of the original antibody, and could guide a functional moiety to the target cell membrane to cause specific cell death without any apparent adverse effects. Further experiments are needed to study the efficacy of this novel mimetic therapy; nevertheless the study provides proof of concept that this novel model of rebuilding antibody molecules offers additional treatment modalities for targeted therapy of solid tumors.

## Competing interests

The authors declare that they have no competing interests.

## Authors' contributions

ZPZ and JZ prepared mimetic and fusion molecules, measured *in vitro *and *in vivo *killing activity and did pathological assays; SYZ did DNA scanning and SDS-PAGE.
